# Progress in investigating the relationship between Schlafen5 genes and malignant tumors

**DOI:** 10.3389/fonc.2023.1248825

**Published:** 2023-09-11

**Authors:** Teng Tu, Ye Yuan, Xiaoxue Liu, Xin Liang, Xiaofan Yang, Yue Yang

**Affiliations:** ^1^School of Basic Medicine, Mudanjiang Medical College, Mudanjiang, Heilongjiang, China; ^2^Beidahuang Industry Group General Hospital, Harbin, China; ^3^The 1st Clinical Medical College, Mudanjiang Medical College, Mudanjiang, Heilongjiang, China

**Keywords:** SLFN 5 gene, tumor growth, tumor metastasis, malignant tumor, review

## Abstract

The Schlafen5(SLFN5)gene belongs to the third group of the Schlafen protein family. As a tumor suppressor gene, SLFN5 plays a pivotal role in inhibiting tumor growth, orchestrating cell cycle regulation, and modulating the extent of cancer cell infiltration and metastasis in various malignancies. However, the high expression of SLFN 5 in some tumors was positively correlated with lymph node metastasis, tumor stage, and tumor grade. This article endeavors to elucidate the reciprocal relationship between the SLFN5 gene and malignant tumors, thereby enhancing our comprehension of the intricate mechanisms underlying the SLFN5 gene and its implications for the progression, invasive potential, and metastatic behavior of malignant tumors. At the same time, this paper summarizes the basis of SLFN 5 as a new biomarker of tumor diagnosis and prognosis, and provides new ideas for the target treatment of tumor.

## 1 Introduction

The increasing incidence of cancer poses a significant threat to human health. While it is well known that the human body possesses a powerful immune system, tumors can proliferate endlessly (1). One characteristic of malignant tumors is their resistance to cell death, specifically resistance to apoptosis (2). Apoptosis is a tightly regulated programmed cell death process characterized by nuclear condensation, cellular shrinkage, and DNA fragmentation (3). Evading cell death is an ability observed in almost all types of cancer cells (4). Signaling molecules responsible for cell apoptosis can be categorized into two groups: pro-apoptotic factors and anti-apoptotic factors. Recent studies have confirmed that DNA damage, imbalances in signaling molecules, and hypoxia may trigger apoptosis in malignant tumor cells (5). During the process of tumor formation, by examining changes in the genomic structure itself, including gene mutations and chromosomal abnormalities, potential factors that influence tumor occurrence, development, and related indicators can be identified. Increasing research results suggest that the Schlafen (SLFN) family plays a role in the immune system and malignant tumors, including melanoma and renal cell carcinoma (6–10). Schlafen was first discovered when it was differentially expressed during thymocyte development and T cell activation in mice (11–13). The term “Schlafen” is derived from the German word meaning “sleep” and refers to the G0/G1 cell cycle arrest observed in ectopic expression of Slfn1 *in situ* in NIH-3T3 fibroblasts (11, 14). The Schlafen family of proteins is a group of genes originally characterized on the basis of their growth-regulatory properties (10, 15). SLFN5 (Human Schlafen 5) is an important molecule in the Schlafen family (16). Studies have shown that human SLFN5 (SLFN5) plays a regulatory role in the proliferation of several specific cancer cells. Firstly, downregulation of SLFN5 promotes the formation of soft agar colonies/non-anchorage-dependent growth in human melanoma cells (17), but does not affect the proliferation of renal cell carcinoma (RCC) cells, and even enhances the growth of glioblastoma cells (18). Therefore, the inconsistent results of SLFN5’s role in different types of tumors have attracted attention, prompting further exploration of its function in other malignancies.

The role of SLFN5 in tumor development may exhibit inhibitory or stimulatory effects depending on the type of tumor. Therefore, further research on the mechanisms of SLFN5 in different types of malignant tumors is of significant importance. Some studies have indicated that SLFN5 is associated with apoptosis and cell cycle regulation in tumor cells. High expression of SLFN5 can promote tumor cell apoptosis and inhibit the cell cycle, thereby suppressing tumor cell proliferation (19). Additionally, SLFN5 can influence tumor cell proliferation and transformation by regulating processes such as DNA replication and repair (20–22). Consequently, SLFN5 may serve as a potential therapeutic target for cancer treatment, with treatment strategies targeting SLFN5 aimed at inhibiting tumor cell proliferation and transformation.

In conclusion, as a member of the Schlafen family, SLFN5 plays an important role in malignant tumors. Although its effects vary across different tumor types, an increasing body of research suggests that SLFN5 may serve as a crucial target for cancer treatment (23). Future studies should continue to investigate the mechanisms and regulatory pathways of SLFN5 in tumors, aiming to provide more effective approaches and strategies for cancer prevention and treatment.

## 2 Structure of the SLFN5 gene and protein

As a member of the third group of SLFNs, SLFN5 shares most of its structural domains with SLFN11, SLFN13, and SLFN14 (20, 21). However, the endoribonuclease activity of SLFN5 appears to be defective, suggesting that SLFN5 has unique functions within the SLFN family (21, 24). The conserved domain COG2865, which is found in known and putative transcriptional regulatory factors and helicases, shows partial homology in SLFN5. Another SLFN-specific domain defined by the sequence Ser-Trp-Ala-Asp-Leu, called the SWADL domain, is present (25). It contains a conserved motif at the C-terminus and is characterized by the presence of sequences homologous to the helicase superfamilies I and UvrD DNA helicases. Additionally, the C-terminal extension carries a nuclear localization signal (RKRRR), further supporting the idea of nuclear functions associated with the Group III SLFNs. SLFN5 possesses a specific SLFN box domain located near the AAA domain (11). Although the “Schlafen box” and SWADL domain are present, their functions remain unknown. In contrast to other SLFNs, SLFN5 does not possess endoribonuclease activity towards tRNA2, although its active site is conserved, suggesting that it may target single-stranded or double-stranded DNA (24, 26). It also contains a nuclear localization signal and predicted DNA/RNA interaction domains.

## 3 SLFN5 protein and its functions

The core structural domain of SLFN5 consists of a positively charged patch, which is in close proximity to the highly conserved zinc finger region proposed for the putative endoribonuclease active site and located at the opposite site of the molecule (21, 24, 25). An assumed zinc finger motif is present on the back valley of SLFN5, suggesting that the putative zinc finger may contribute to the recognition of nucleotide targets or assist in protein folding and the endonuclease/ATPase activities within the M and C domains (20). The M domain of SLFN5 exhibits a twist where it connects to the assumed helicase/ATPase in the C domain (20). The SLFN family has been found to play a crucial role in tumor development and drug resistance. High expression of SLFN5 in melanoma (6), renal cell carcinoma (27), and breast cancer inhibits tumor invasion and migration (28), indicating that SLFN5 acts as a tumor suppressor gene. However, elevated SLFN5 expression in glioblastoma, pancreatic ductal adenocarcinoma, and prostate cancer promotes tumor proliferation, invasion, and metastasis (21). Nevertheless, the expression of SLFN5 is still induced by interferon (IFN), and IFN-activated SLFN5 is primarily localized in the cell nucleus and inhibits anchorage-dependent growth of melanoma cancer cells (6). SLFN5 downregulates membrane type 1 matrix metalloproteinase (MT1-MMP) expression through the AKT/GSK-3β/β-catenin pathway in several types of cancer cells, exerting inhibitory effects on migration and invasion (17). SLFN5 also negatively controls STAT1-mediated transcriptional activation of IFN-stimulated genes and *ZEB1* transcription, suppressing the antitumor immune response in glioblastoma cells and the mesenchymal-epithelial transition (18, 29). Low expression of the SLFN5 protein is significantly associated with various clinical-pathological variables, including tumor diameter, T classification, N classification, and clinical staging.

## 4 Association between SLFN 5 and malignant tumors

### 4.1 SLFN5 and breast cancer

**Figure 1:**
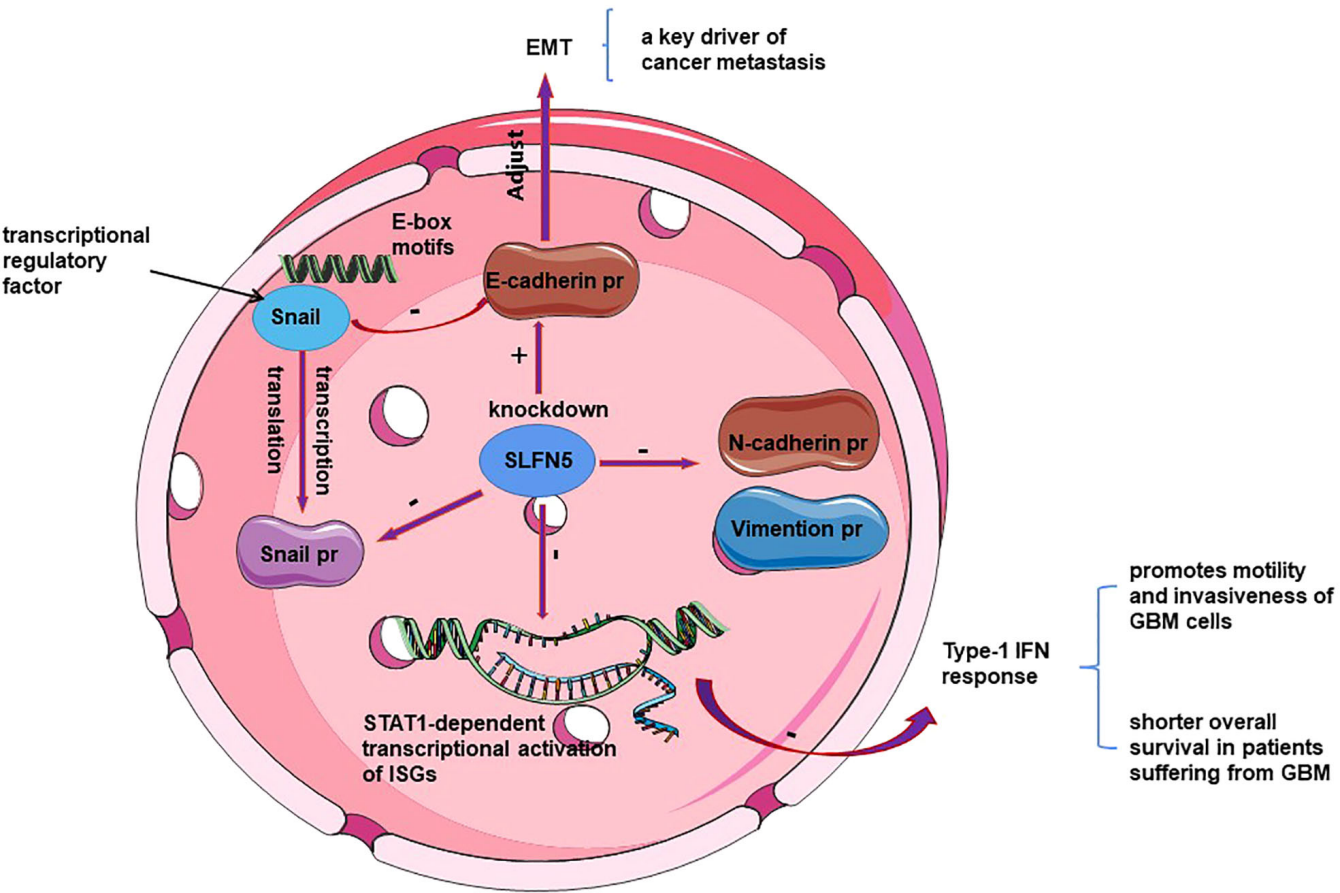
Schematic representation of the mechanism by which SLFN 5 regulates BRCA progression.

As a transcriptional repressor, SLFN5 prevents epithelial-mesenchymal transition (EMT) in breast cancer and targets the ZEB1 promoter to suppress ZEB1 transcription and downstream PTEN/AKT/cyclin D1 signaling cascade, ultimately inducing cancer cell death (28, 29). Through differential expression analysis using data from The Cancer Genome Atlas (TCGA), clinical samples, and cell lines, Gu et al. (28) found a negative correlation between SLFN5 expression and breast cancer metastasis. Wan et al. (29) 8demonstrated, through knockdown and overexpression of SLFN5, as well as luciferase reporter gene assays and metabolomics analysis, that SLFN5 regulates PTEN transcription, the AKT pathway, and proliferation/apoptosis through ZEB1 mediation, and inhibits purine metabolism, leading to cancer cell death. SLFN5, as a transcriptional repressor (30), plays a crucial role in various cancers. In breast cancer, downregulation of SLFN5 protein in breast cancer cells increases ZEB1 transcriptional activity. Subsequently, upregulated ZEB1 protein binds to the PTEN promoter, inhibiting PTEN expression, activating the AKT pathway, and promoting breast cancer progression (28) (see **Figure 1**). Thus, SLFN5 suppresses cancer cell proliferation and induces apoptosis by regulating the ZEB1/PTEN/AKT pathway and purine metabolism (17, 28). PTEN is considered a tumor suppressor gene that can dephosphorylate phosphatidylinositol-3, 4, 5-triphosphate (PIP3) to PIP2, thereby antagonizing phosphoinositide-3-kinase (PI3K) signaling and AKT phosphorylation/activation, which in turn affects cell cycle progression, apoptosis and motility (31–33). Notably, knockdown of ZEB1 led to induction of PTEN with loss of constitutive pS473Akt (34). Additionally, it maintains and restores the epithelial morphology of breast cancer cells by downregulating ZEB1 transcription, thereby preventing EMT in breast cancer. Li et al. (35), through bioinformatics analysis of TCGA data, discovered that miR-146b-5p can bind to the SLFN5 3’UTR and MEG3, demonstrating that MEG3 positively regulates SLFN5 expression and inhibits breast cancer development through sequestration of miR-146b-5p (see **Figure 1**). MEG3 is downregulated in various cancers and positively correlates with SLFN5 expression in breast cancer (36, 37).

### 4.2 SLFN5 and gastric cancer

Currently, there is limited research on the role of SLFN5 in gastric cancer (GC) (38). Xu et al. (13) investigated the relationship between SLFN5 expression and tumor stage, lymph node metastasis, and tumor grade using the UALCAN database. The results revealed a positive correlation between high expression of SLFN5 in GC and lymph node metastasis, tumor stage, and tumor grade. The functional implications of SLFN5 were also explored. Comparative analysis using the TCGA database demonstrated significantly higher expression of SLFN5 in gastric tumor tissues compared to normal tissues, suggesting that SLFN5 may promote the development of gastric cancer. KM plotter analysis showed that high expression of SLFN5 was associated with poor prognosis, specifically lower overall survival (OS) and progression-free survival (PFS), indicating that SLFN5 could serve as a prognostic indicator for this disease. KEGG pathway enrichment analysis of SLFN5 revealed its involvement in T-cell activation and immune response regulation. Specifically, SLFN5 expression in GC showed a significant positive correlation with infiltration of CD8 T cells, CD4 T cells, macrophages, neutrophils, and dendritic cells. Additionally, SLFN5 expression exhibited a positive association with natural killer (NK) cells, Th17 cells, and regulatory T (Treg) cells in GC. Tsao AC et al. (39) found that SLFN5 co-localized with T cells and M2 macrophages in gastric cancer precancerous lesions, suggesting an immune suppressive role of SLFN5 in GC. SLFN5 was predominantly expressed in Treg and naive T cells, which play a tumor-promoting role in cancer (40, 41). Intestinal metaplasia (IM) is the highest-risk precursor lesion for gastric cancer (GC) progression. Companioni Napoles et al. (39) observed the highest levels of SLFN5 expression in IM subjects progressing to GC through immunohistochemical detection and statistical analysis. Thus, elevated SLFN5 protein expression in IM subjects was associated with gastric cancer progression.

### 4.3 SLFN5 and pancreatic ductal adenocarcinoma

Fischietti et al. (42) inferred that high expression of the human SLFN5 gene is associated with poor clinical prognosis in patients with pancreatic ductal adenocarcinoma (PDAC), indicating the involvement of SLFN5 in the occurrence and progression of PDAC, leading to adverse outcomes. Furthermore, Frank et al. conducted additional research and found a positive correlation between SLFN5 expression and PDAC grading, depth of infiltration, lymph node metastasis (LNM), and TNM staging. They also performed cell viability assays based on Alamar Blue and observed a significant reduction in tumor cell viability upon SLFN5 depletion. Through in-depth analysis, SLFN5 was identified as a novel stimulator of cell cycle progression in the S phase, mediating interaction with E2F7 and regulating potential genes involved in cell cycle progression, thereby slowing down the process. Felix et al. (43) demonstrated a significant increase in SLFN5 expression at both the mRNA and protein levels upon ZNF154 transfection. Thus, it is suggested that SLFN5 may play a role in pancreatic cancer and is associated with the upregulation or downregulation of the ZNF154 gene. Increased survival rates in pancreatic cancer patients were associated with the silencing of ZNF154, which, in turn, led to increased levels of SLFN5. However, the precise mechanism between ZNF154 and SLFN5 still requires further clarification (44).

### 4.4 SLFN5 and glioblastoma

**Figure 2:**
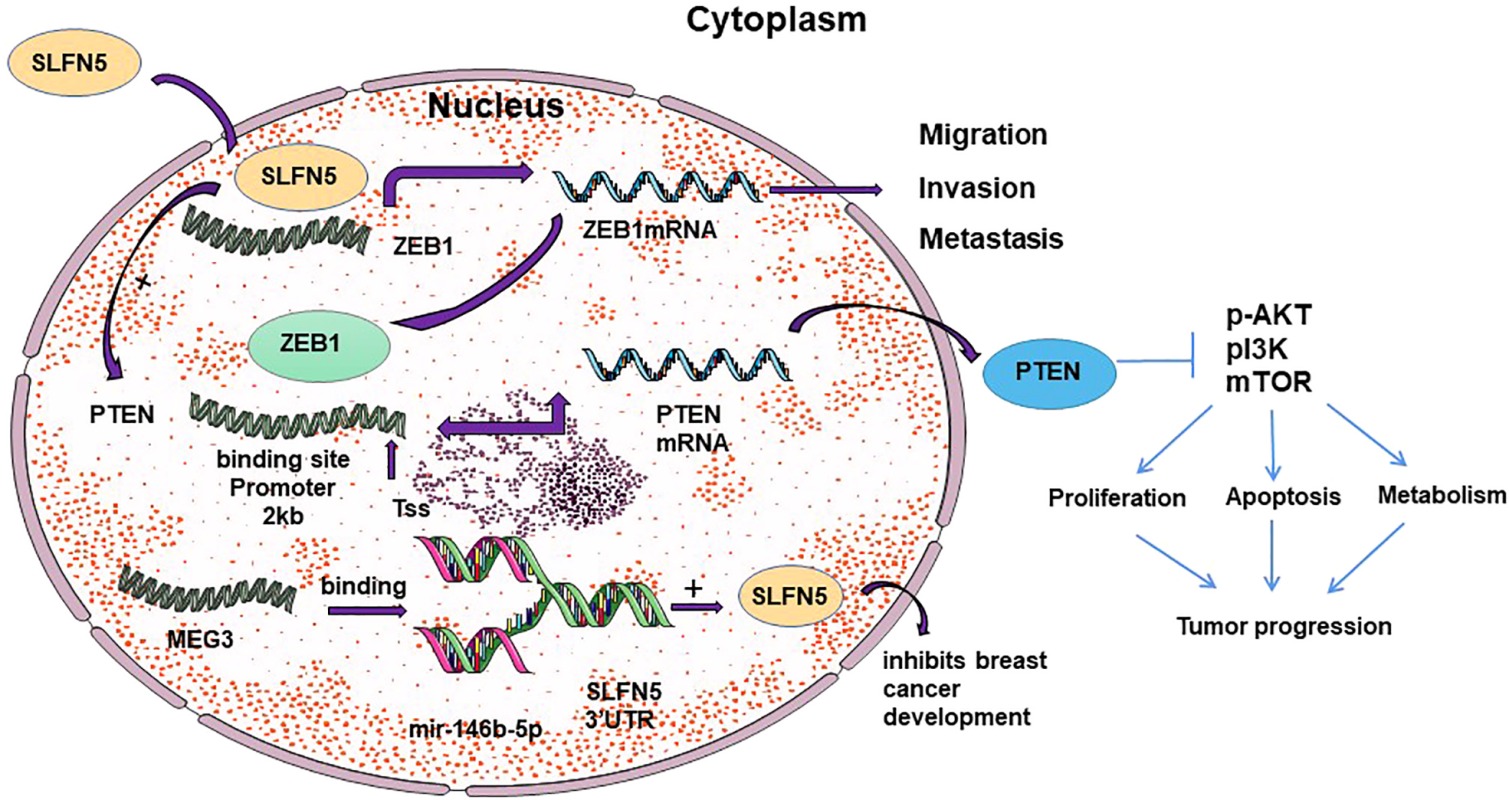
Schematic representation of the mechanism of SLFN 5 regulating ovarian cancer and glioblastoma.

SLFN5 plays different roles in specific types of cancer cells through distinct mechanisms, exhibiting inhibitory effects in some cancers while promoting effects in others. However, the specific mechanisms underlying these roles are not yet fully understood. Ahmet et al. (18) conducted a study demonstrating that SLFN5 expression promotes the motility and invasiveness of glioblastoma multiforme (GBM) cells, and high levels of SLFN5 expression are associated with lower survival rates in high-grade gliomas and GBM patients. Protein analysis revealed overexpression of SLFN5 in malignant brain tumor cells. High expression of SLFN5 facilitates the proliferation, invasion, and *in vivo* growth of GBM cells. SLFN5 can be induced by type I interferon (IFN) in malignant brain tumor cells and acts as an inhibitor of gene transcription driven by STAT1 through direct protein interactions (18, 45, 46). The enrichment of SLFN5 binding sites within the promoter of STAT1-induced type I IFN-stimulated genes suggests that SLFN5 functions as a negative regulator of the overall transcriptional response to IFNβ (47, 48) (See **Figure 2**). Targeting SLFN5 may serve as a promising approach for the treatment of drug-resistant glioblastoma and/or the elimination of glioma cancer stem cells, but further research is needed to explore its feasibility and effectiveness (49).

### 4.5 SLFN5 and renal cell carcinoma

Renal cell carcinoma (RCC) is highly sensitive to interferon treatment (50). Sassano et al. (27) demonstrated that the expression of human SLFN5 can be induced by type I interferon receptor and proposed an inverse relationship between SLFN5 expression and the invasiveness and motility of renal cell carcinoma. SLFN5 inhibits the motility and invasiveness of malignant renal cell carcinoma cells by negatively controlling the expression of matrix metalloproteinase genes (such as MMP-1 and MMP-13). T-test analysis of RCC samples revealed a positive correlation between higher levels of SLFN5 expression and overall survival rates in RCC patients. This indicates that SLFN5 possesses tumor-suppressive activity. In renal cell carcinoma, SLFN5 is also associated with clinical prognosis. Liu et al. (51) determined the expression level of SLFN5 protein in renal clear cell carcinoma using immunohistochemical techniques and found that SLFN5 expression decreased with increasing age, higher pathological grade, higher T stage, and clinical stage in RCC patients. Kaplan-Meier analysis showed that patients with high expression of SLFN5 protein had longer overall survival time and disease-free survival time compared to those with low expression. Furthermore, multivariate Cox regression analysis revealed that patients with high expression of SLFN5 protein had significantly longer overall survival time and disease-free survival time compared to those with low expression. Therefore, SLFN5 has the potential to serve as a novel clinical indicator for the prognosis of renal cell carcinoma (RCC).

### 4.6 SLFN5 and lung cancer

SLFN5 represents a promising biomarker for early-stage non-small cell lung cancer (NSCLC) patients (52). The downregulation of SLFN5 is strongly associated with disease progression and poor prognosis in NSCLC. Studies have consistently shown weak expression of SLFN5 in NSCLC tissues. The reduced expression of SLFN5 protein is significantly correlated with several clinical and pathological variables, including tumor diameter, T classification, N classification, and clinical staging. Notably, NSCLC patients with high expression of SLFN5 protein demonstrate significantly improved overall survival rates. Epithelial-mesenchymal transition (EMT) is a critical initial step for tumor cells to acquire invasive and metastatic capabilities. Gu et al. (53) conducted comprehensive experiments utilizing green fluorescent protein labeling, wound healing assays, real-time quantitative PCR, and protein blotting. The results unveiled that SLFN5 overexpression promotes EMT in lung cancer cells. As EMT progresses, lung cancer cells exhibit increased nuclear or cytoplasmic translocation of β-catenin, elevated levels of the EMT-related transcription factor Snail, and decreased expression of E-cadherin, ultimately leading to enhanced cell invasion and migration. These findings indicate that heightened expression of SLFN5 augments the migration and invasion capabilities of lung cancer cells. SLFN5 promotes EMT and cell metastasis in human lung cancer cell line A549 by inducing translocation of β-collagen from the cell membrane to the nucleus, thereby activating the β-collagen-mediated snail/E-calmodulin signaling pathway (30).Wan et al. identified a critical role for SLFN5 in maintaining non-/low-invasive cancer cell lines (breast cancer cell line MCF5, colorectal cancer cell line HCT7, and lung cancer cell line A116) in a non-invasive state, and found that the β-cartenin pathway mediated SLFN5 on MT1-MMP expression (17). In lung cancer, PTEN is regulated by p53 (54, 55), Oct4 (56), c-Jun (57), and NF-κB (58). Regarding signaling pathways, human SLFN5 exerts inhibitory effects on lung cancer progression through the PTEN/PI3K/AKT/mTOR pathway. Collectively, the data strongly suggest that SLFN5 may play a crucial role in improving the prognosis of lung cancer patients and has the potential to serve as a valuable biomarker for predicting patient outcomes.

### 4.7 SLFN5 and malignant melanoma

Due to the dependency of SLFN5 protein on I-type interferon (IFN) (21), it is well-established that SLFN5 plays a critical role in the inhibitory effects of IFNα on the growth and invasion of malignant melanoma cells. Notably, stable suppression of SLFN5 expression in these cells fails to optimally achieve the inhibitory effects of IFNα. The induction of human SLFN5 by human IFNα in malignant melanoma cells suggests its specific involvement in the IFNα response pathway. Enhancing the inhibitory effects of SLFN5 expression in malignant melanoma cells may confer a growth advantage and promote tumor development. Katsoulidis et al. (6), employing soft agar assays and enhanced colony formation, demonstrated that stable knockdown of SLFN5 in malignant melanoma cells leads to increased anchorage-dependent growth. Additionally, collagen invasion plays a crucial role in melanoma progression, and the knockdown of SLFN5 also results in augmented invasion of three-dimensional collagen, indicating that SLFN5 has a dual role in regulating the invasion and anchorage-independent growth of melanoma cells.

### 4.8 SLFN5 and castration-resistant prostate cancer

Mark et al. (23), employing comparative proteomic analysis, discovered SLFN5 as a protein regulated by the androgen receptor (AR) in castration-resistant prostate cancer (CRPC). Mechanistically, SLFN5 interacts with ATF4 and modulates the expression of LAT1, a crucial amino acid transporter. Consequently, depletion of SLFN5 in prostate cancer (CRPC) cells diminishes the levels of intracellular essential amino acids and disrupts mTORC1 signaling in a LAT1-dependent manner. This investigation identifies SLFN5 as a novel regulator of the LAT1 amino acid transporter and a significant contributor to mTORC1 activity in castration-resistant prostate cancer. Immunohistochemical analysis of prostate cancer (CRPC) specimens unveiled a noteworthy association between SLFN5 expression and disease progression, along with a substantial correlation with an elevated risk of metastasis.

### 4.9 SLFN5 and ovarian cancer

It is widely acknowledged that ovarian cancer cells have a propensity for metastasis. Epithelial-to-mesenchymal transition (EMT) is a crucial step in the metastatic process, facilitating the detachment of tumor cells from the primary site and their adhesion to secondary sites (59). Xu et al. (60) investigated the role of the slfn5 gene in ovarian cancer epithelial-mesenchymal transition (EMT) and its influence on tumor invasion and migration by silencing the slfn5 gene. They observed high expression of the slfn5 gene in human ovarian cancer cell lines. The study demonstrated that silencing the SLFN5 gene led to reduced expression of EMT-related proteins in ovarian cancer cell lines. Specifically, it resulted in decreased levels of N-cadherin and Vimentin, increased levels of E-cadherin, and decreased levels of Snail protein. Notably, when the Snail transcription factor binds to the E-box motif (61), it causes a reduction in E-cadherin expression (see **Figure 2**). E-cadherin, an EMT-related protein, is a characteristic protein involved in mediating cell-cell interactions within the epithelial phenotype (62). N-cadherin and Vimentin proteins are often used as tumor markers for identifying the mesenchymal phenotype (63). SLFN5 expression levels increase with the malignant tumor grade and reach their peak in ovarian tumors (13). Moreover, elevated levels of SLFN5 expression are significantly associated with poor prognosis in ovarian cancer patients, and targeting SLFN5 has been found to inhibit ovarian tumor growth both *in vitro* and *in vivo (* 60). However, the observed anti-tumor effects resulting from SLFN5 depletion are partially attributed to its interference with cell cycle progression. This suggests that cell cycle dysregulation is a distinct characteristic of ovarian tumors, as well as several other types of cancers (60, 64). Consequently, SLFN5 has been identified as a novel promoter of S-phase progression, potentially broadening the repertoire of available cell cycle inhibitors (65, 66).

## 5 Conclusions

In conclusion, the research on the regulatory mechanism of SLFN5 expression has achieved good results in recent years, such as the breakthrough in the study of the pathway through which SLFN5 affects tumor development, the proposed inhibition of MT3-MMP expression by SLFN5 through the AKT/GSK-3β/β-catenin pathway to inhibit the migration and invasion of cancer cells, and SLFN 5 regulation of LAT 1-mediated mTOR activation in depot resistant prostate cancer regulates LAT 1-mediated mTOR activation, among other findings. More convincing studies have been carried out at affecting reversible epithelial and epithelial-mesenchymal transition; and research ideas have been clarified in studying the effects of SLFN 5 on different tumors. However, it cannot be ignored that there are still a large number of problems to be solved in the study of SLFN5, such as the lack of research on the regulatory mechanism and effect of SLFN5 in gastric cancer and glioblastoma, and the lack of research on the effect of SLFN5 as a biomarker of chemotherapy and a potential target of antitumor drugs in the clinic, on the one hand, it is because the effect of SLFN5 is not universal, and has not the same effect on different types/subtypes of cancers, on the other hand, it is not universal. On the one hand, SLFN5 is not universal, and has different mechanisms of action for different types/subtypes of cancers and different effects on cancers, and part of the mechanism of SLFN5 has not yet been clarified and is in the early stage of research; on the other hand, there is no clear research that can show whether SLFN5 is easy to mutate and whether it is stable when put into the production of drugs.

## Author contributions

TT wrote the manuscript. YYu and YYa designed and supervised the study. XL provided critical suggestions for the manuscript and revised the revised manuscript. XXL revised the manuscript. XY revised the manuscript and supervised the study. All authors contributed to the article and approved the submitted version.
